# Evaluation of a facilitator training program in a randomized controlled trial of psilocybin treatment for depression

**DOI:** 10.1186/s12909-026-09124-8

**Published:** 2026-04-09

**Authors:** Nikita Morel, Dea Siggaard Stenbaek, Johan Lundberg, Maria Beckman

**Affiliations:** 1 Department of Psychology, Copenhagen University Clinic for Psychedelic Research, University of Copenhagen, Copenhagen, Denmark; 2 Neurobiology Research Unit, Copenhagen University Hospital, Copenhagen, Denmark; 3 Centre for Psychiatry Research, Department of Clinical Neuroscience, Karolinska Institutet and Stockholm Health Care Services, Region Stockholm, Stockholm, Sweden

**Keywords:** Psilocybin, Psychedelic, Cancer-Related Depression, Set and Setting, Psychotherapeutic Support, Facilitator Training, Training Evaluation, Nurses

## Abstract

**Background:**

Major depression is a prevalent condition among patients with life-threatening illnesses, such as cancer, and recent findings suggest that psilocybin may hold promising treatment potential. Contemporary trials of psilocybin generally employ a model that includes psychotherapeutic support consisting of preparation and integration sessions surrounding the dosing. However, there is limited research on the psychotherapeutic component of treatment, including the skills, professional qualifications and training needed to provide it.

**Methods:**

In this study, nine nurses completed a 15-week online and on-site training program as facilitators in an ongoing randomized controlled trial of psilocybin treatment for depression. The training evaluation consisted of a subjective evaluation by the facilitators collected during and after completion of training, and an objective evaluation of the facilitators’ verbal relational skills assessed with standardized role-plays before and after completion of training. The recorded role-plays were assessed using the relational components of the Motivational Interviewing Treatment Integrity (MITI) code 4.2 and analyzed with the Wilcoxon Signed Rank Test.

**Results:**

The facilitators’ subjective evaluations indicated that the online and on-site training sessions had supported their knowledge- and skill acquisition. However, most facilitators reported that additional practical in-person training would have been necessary for them to feel adequately prepared to provide the treatment. The objective assessment of the facilitators’ verbal relational skills showed a significant increase in one of twelve MITI variables and medium to large effect sizes for six of the measures pre- to post-training.

**Conclusions:**

The training model used in this study showed potential to improve outcomes, though effects were modest and only demonstrated in role-play. The facilitators also indicated a need for additional training to feel adequately prepared. The exact requirements for the psychotherapeutic support surrounding the dosing in these treatments, including the specific skills and professional qualifications needed to provide it, remain unclear. Nonetheless, the results of this study suggest that different professionals may require distinct types of training to deliver these treatments effectively. Future studies should design training programs based on the facilitators’ baseline skills and provide clear descriptions and objective measures of both the training intervention and outcome, along with adherence measures throughout treatment.

**Trial registration:**

EudraCT: 2023-505532-35-00; Clinicaltrails.gov ID: NCT06319378. Registered 8 November 2023.

**Supplementary Information:**

The online version contains supplementary material available at https://doi.org/10.1186/s12909-026-09124-8.

## Background

Major depression is a prevalent condition among patients with life-threatening illnesses, such as cancer, affecting an estimated 13% to 27% of individuals [1, 2]. Furthermore, cancer-related depression is associated with poor treatment adherence, shortened survival time, lower quality of life and increased risk of suicide [3–5]. Current treatment options for depression, including pharmacotherapies and psychotherapeutic interventions, have proven insufficient for a significant number of patients and novel approaches are increasingly being researched. Recent findings suggest that treatments utilizing classic psychedelics, such as psilocybin, may offer promising potential for the treatment of depression [6], particularly in patients with life-threatening illnesses [7–10].

Psilocybin is a naturally occurring psychedelic compound that can produce an altered state of consciousness, characterized by profound changes in affect, perception, and cognition [11]. Research has demonstrated that the acute effects of psilocybin are highly influenced by the contextual factors surrounding its administration, commonly referred to as set (i.e., mindset and psychological disposition) and setting (i.e., physical, social and cultural setting) [12]. The conditions under which psilocybin is administered are therefore carefully managed across most contemporary clinical trials, where set and setting protocols are employed to ensure patients’ safety and wellbeing [13]. Clinical trials typically employ a model that includes psychotherapeutic support consisting of preparation and integration sessions surrounding the dosing [14]. This places psilocybin treatments at the intersection of pharmacological and psychotherapeutic interventions. However, contemporary psilocybin research has primarily focused on the clinical efficacy of the pharmacological component, while research in the psychotherapeutic component remains scarce. As such, best practices regarding the psychotherapeutic support in psilocybin treatments, including which professions should provide it and how they should be trained, have yet to be established [15]. It is also worth noting that in the broader field of psychedelic research, some researchers are proposing models that minimize psychotherapeutic support [16], representing a substantial divergence from trials emphasizing comprehensive facilitator-led interventions.

The psychotherapeutic support has varied considerably across trials, reflecting the lack of consensus on what this component should entail to be sufficient and effective. Additionally, it is rarely described in accordance with the standards of clinical research [17]. In a recent review of the psychotherapeutic support in psilocybin treatments for depression, the therapeutic framework ranged from nondirective support models to various evidence-based methods, with the number of treatment sessions ranging from five to ten. The personnel delivering the psilocybin treatment, here referred to as facilitators, had diverse professional backgrounds, consisting primarily of psychologists and psychiatrists, with occasional inclusion of nurses, social workers, and counselors [18]. These variations in structure, content, and the professional backgrounds of facilitators delivering these treatments, combined with the insufficient reporting in contemporary clinical trials, make it difficult to determine the specific skills required to effectively provide these treatments. Still, due to the unpredictable and often vulnerable nature of psychedelic experiences, relational skills – widely recognized as critical in traditional psychotherapy [19–22] – have been suggested as essential in psilocybin treatments regardless of facilitators’ professional backgrounds or the treatment model utilized [23]. One way of measuring relational skills is with the relational components of the Motivational Interviewing Treatment Integrity (MITI). MITI is a behavioral coding system developed to measure therapists’ motivational interviewing (MI) skills in training, clinical treatment and research [24], but it can also be used more broadly as a measure of therapists’ verbal relational skills in all areas of clinical practice. The measure reflects core principles of client-centered and humanistic psychology [25], which are traditionally recognized as important in psychedelic treatments [26]. Enhancing MITI-rated skills, such as empathy and partnership, may be impactful in psilocybin treatments, where a strong therapeutic alliance has been shown to predict both acute effects and clinical outcomes [27].

Another key factor recognized in traditional psychotherapy is the importance of therapist training to develop the skills and competencies needed to deliver psychotherapeutic interventions in clinical trials [28]. In the psychedelic research field, however, most clinical trials include no reports of the methods of training employed [17] and a comprehensive description of a training program for a psilocybin treatment in a clinical trial can only be found in one publication to date [29]. Extensive research has shown that therapist training can effectively improve clinical skills and competencies [30, 31], including relational variables [32], with several reviews specifically highlighting the importance of including active training components and supervision [33–36]. Although more intensive models with multiple training components appear most promising, they also tend to be more costly and resource-intensive. To meet these challenges, online formats are used increasingly and show promising effectiveness [31, 37]. However, there is still some debate about whether more complex treatments are best taught in-person [36]. In the aforementioned publication on the training program for a psilocybin treatment by Tai and colleagues (2021), the training program comprised multiple components and was delivered in a hybrid format combining in-person and online training. While it received positive evaluations from the trainees, no objective measures of training effectiveness were employed in the study.

This study aims to increase knowledge of how facilitators can effectively be trained to provide psychotherapeutic support in psilocybin treatments. It describes and evaluates the training of nurses as facilitators in an ongoing Swedish clinical trial with psilocybin for cancer-related depression, including their subjective evaluations of the training program and an assessment of whether it improves their verbal relational skills.

## Method

This study describes and evaluates the training of nurses as facilitators in the ongoing clinical trial CAPSI: Cancer-related major depression treated with a single dose of psilocybin. The training program was conducted online and on-site in Stockholm, Sweden during October 2023-January 2024. The evaluation consisted of two parts: a subjective training evaluation by the facilitators collected both during and after completion of training, and an objective evaluation of the facilitators’ verbal relational skills assessed with standardized role-plays before and after completion of training.

### The CAPSI trial

CAPSI is a multicenter, randomized (2:1), double-blind, placebo-controlled phase II trial (EudraCT: 2023-505532-35-00; Clinicaltrails.gov ID: NCT06319378) comparing the effect of a single oral dose of 25 mg psilocybin and psychotherapeutic support, with the effect of a single oral dose of an active placebo (1 mg psilocybin) and psychotherapeutic support for cancer-related major depression. Recruitment for the CAPSI trial started in March 2024 and a total of 100 patients with cancer and depression will be recruited across four sites in Sweden during 2024–2025. The primary end point is depression severity measured with Montgomery & Åsberg Depression Rating Scale (MADRS) six weeks after dosing. Follow-up assessments include psychiatric evaluation in addition to online symptom rating of depression up to six months after dosing and several other measures evaluating medical, therapeutic and health economic aspects of psilocybin treatment for depression in cancer patients. All treatment facilitators are trained nurses. The treatment consists of one dosing session, two preparation sessions and three integration sessions. Preparation sessions are held two weeks and one week prior to the dosing session. The first integration session takes place within the first days after the dosing session, with the second and third sessions scheduled one week apart. Each preparation and integration session lasts approximately 90 min and is conducted by the same facilitator in the dosing room, apart from the first preparation session, which is conducted online. Psilocybin is only administered during the dosing session, which lasts seven to nine hours. During this session a nurse assistant is present alongside the facilitator.

### Participants

The participants in this study consisted of the nine facilitators in the CAPSI trial. Their mean age was 37.4 (*SD* = 5.1) and six of them were female. All of them were employed at the four sites and they had several years of work experience as registered nurses (*M* = 13.1, *SD* = 5.7). Eight of the facilitators had a bachelor’s degree and one had a master’s degree. Five of them had additional specialist training in psychiatry, one in intensive care, and one in anesthesia. Additionally, one of the facilitators had basic training in psychotherapeutic methods.

### Training program

**Fig. 1:**
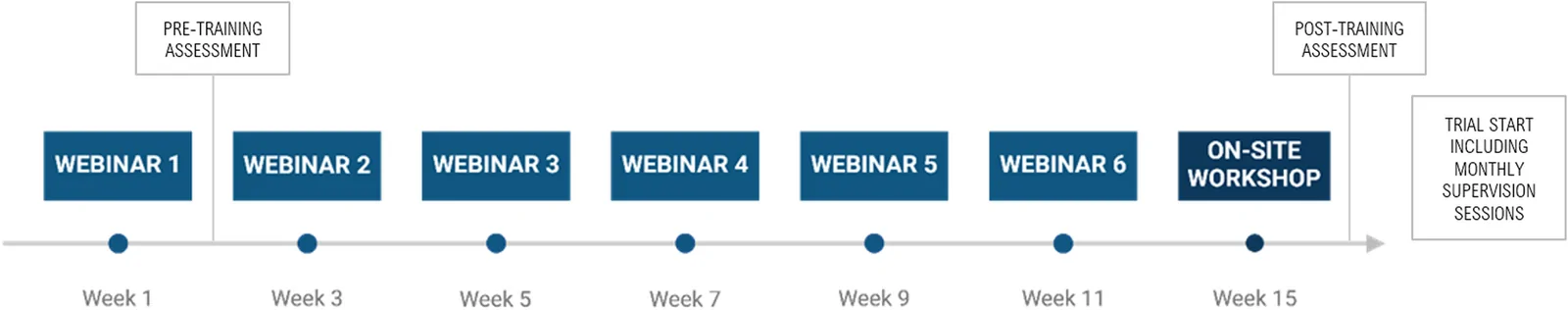
Timeline of the facilitator training program

The training program was designed and conducted by two of the researchers responsible for the therapeutic component and treatment manual in the CAPSI trial (authors: DSS and MB). The training consisted of two components: online training comprising six biweekly webinars lasting three hours each, followed by a three-day on-site training workshop lasting 19 h in total. The full training spanned 15 weeks (Fig. 1). Throughout the entire training period, the facilitators had access to an online platform containing all relevant training materials.

Each of the six webinars focused on a specific theme of content and included lectures delivered by the primary instructors (DSS and MB) and guest lecturers specializing in selected areas of treatment, as well as practical exercises and group discussions. The facilitators were also provided with reading materials on the topics covered to prepare for each session.

The first webinar included a general introduction to psilocybin, the CAPSI trial, the target population and the treatment protocol. A guest lecturer covered the topic of anxiety and depression in patients diagnosed with cancer. The second webinar introduced therapeutic communication techniques and verbal relational skills, as well as the preparation sessions of treatment and the patient exercises. Training in these areas continued throughout the third webinar. The fourth webinar addressed the dosing session with particular focus on the role of music in psilocybin treatments, with a guest lecturer covering music and altered states of consciousness, the Copenhagen Music Program [38], and the Guided Music Visualization and free Association Exercise (GMVA) utilized in treatment. The fifth webinar included a review and practice of all the patient exercises and therapeutic communication techniques. The sixth and final webinar covered both the dosing and integration sessions of the treatment. During this webinar, facilitators were also provided with a practical roadmap of the full treatment, with time allocated for discussing the therapy outline and standard operating procedures.

The subsequent three-day on-site training workshop was conducted during an extended weekend (i.e., Friday to Sunday) at Karolinska Institutet. It was led by the primary instructors, DSS and MB, joined by music and GIM-therapist Catharina Messel, and focused on revising and applying the material covered during the webinars, with in-depth training of the treatment components.

### Measures

#### Facilitators’ training evaluation

The subjective training evaluation was developed for this study and conducted anonymously by the facilitators in three parts (supplementary 1): At the beginning of the on-site training, they were first asked to provide an evaluation of the online training in free text, detailing their perceived learning outcomes from the webinars and identifying areas where they felt further training was necessary. On the final day of the on-site training workshop, they were asked to provide a second evaluation, this time of the on-site training and their perceived progression. Finally, one week after the end of the full training period, and before commencement of the trial, the facilitators completed an online survey evaluating the complete training program by rating 13 items on a Likert-scale from one (*No*,* definitely not*) to five (*Yes*,* definitely*) (Table 2), with five additional questions in free text format.

#### Assessment of facilitators’ verbal relational skills

To evaluate the facilitators’ verbal relational skills before and after completion of training, the facilitators were asked to conduct two standardized role-plays of patient phone calls. Both role-plays modeled the first patient-contact in the CAPSI trial, specifically the process of booking the patient for their first preparation session. The actor role-playing as patient followed a structured scenario that outlined the role, emotional tone, and key prompts, including sounding worried and asking multiple questions about the treatment. Each role-play lasted approximately 20 min. The first role-play assessed the facilitators’ verbal relational skills pre-training and was set after webinar one but before webinar two, when they were introduced to the therapeutic communication techniques. The facilitators were given the basic instructions to act as they normally would when speaking with a patient. The second role-play, assessing the facilitators’ verbal relational skills post-training, was set after completion of the entire 15-week training program and before commencement of the trial. The facilitators were then given the instruction to apply their verbal relational skills as learned during training when speaking with the patient.

The standardized role-plays were recorded and coded with the relational components of the Motivational Interviewing Treatment Integrity (MITI) code 4.2 (Swedish version) by two coders at The Motivational Interviewing Coding (MIQA) Lab at Karolinska Institutet in Sweden. The instrument includes two components: four global scores and ten behavior counts. In this study, only the ten behavior counts and the two global scores specifically pertaining to therapists’ relational skills were included: (1) *Partnership*, which measures the extent to which the therapist conveys an understanding of the patient as an agent of change. Rather than assuming an expert role, the therapist facilitates a conversation between two equal partners, who both have the necessary knowledge and wisdom to collaborate in creating the desired change. The therapist may do this successfully through genuine negotiation, curiosity and privilege of the patient’s input, as well as emphasis on the patient’s resources and strengths. (2) *Empathy*, which measures the extent of the therapist’s effort to understand the patient’s experience and perspective, and their ability to convey this understanding to the patient through behaviors such as insightful questions and reflections [39]. The global scores are rated on a scale of one to five, with five representing the highest rating. According to the current MITI guidelines, the suggested basic competence and proficiency threshold for therapists’ relational variables (i.e., *Partnership* and *Empathy*) are 3.5 indicating a fair level of skills and 4.0 indicating a good level of skills [39]. Additionally, in this study, all ten MITI behavior counts were included, assessed by counting all instances of specific therapist utterances, with the final score being the tallied count. The ten categories of therapist behaviors are: *Affirm*, *Seeking collaboration*, *Emphasizing autonomy*, *Persuade*, *Confront*, *Giving information*, *Persuade with permission*, *Simple reflection*, *Complex reflection*, *Question* [39]. Consistent with MIQA coding standards, all coders complete extensive training consisting of 120 h of instruction and participate in weekly group coding sessions. To maintain consistent inter-rater reliability in MITI coding, coders also double-code 12 randomly selected recordings twice a year. The MIQA coders are independent of this study and were not involved in the training or clinical trial. To ensure reliability, they were also blinded to the pre/post status.

### Data analyses

The inter-rater reliability of the MITI coding, executed by the MIQA coders, were estimated by calculating the intraclass correlation coefficients (ICC), assessed with a two-way mixed model with absolute agreement, single measures (Table 1). The ICCs are interpreted according to the following recommendations of Koo & Li (2016): < 0.50 *poor*; 0.50 to 0.75 *moderate*; 0.75 to 0.90 *good*; >0.90 *excellent* [40]. An ICC may be reported as 0 if a given behavior occurs so infrequently that a meaningful estimate of agreement cannot be calculated. The qualitative data gathered from the training evaluation was sorted into themes to identify common points of feedback. Themes were developed inductively and compared until consensus was reached by two researchers to ensure reliability, before being summarized in text. Descriptive statistics were generated and presented as frequency, mean, median, standard deviation and interquartile range. Due to data not following a normal distribution, a Wilcoxon signed-rank test was used to assess the change in facilitators’ verbal relational skills from pre- to post-training. The Benjamini–Hochberg procedure was applied as a correction for multiple comparisons, and the effect sizes were calculated using Pearson’s correlation coefficients: *r* < .10 small; *r* < .30 medium; *r* < .50 large [41]. All quantitative data in the study were analyzed using the Statistical Package for the Social Sciences (SPSS), version 22.0.

**Table 1:** The intraclass correlation coefficients of the MITI codings

MITI variable	*ICC*
Relational Global	.84
Total MI-adherent	.88
Giving information	.92
Persuade with permission	0
Simple reflection	.92
Complex reflection	.91
Question	.96

*ICC *Intraclass correlation coefficients; Relational Global = *Partnership* and *Empathy*; Total Ml-adherent = *Seeking collaboration*; *Affirm* and *Emphasizing autonomy*

## Results

### The facilitators’ subjective training evaluation

The 13-item training evaluation is summarized in Table 2. In the free text evaluations, a majority of the facilitators expressed that the webinars had given them a general overview of the study, knowledge of important treatment components such as psilocybin, a better understanding of their role as facilitators and practice of therapist skills and behaviors.

**Table 2:** The 13-item (1–5) subjective training evaluation completed post-training by the CAPSI facilitators

Item	*N*	*M* ± *SD*	Range: min, max
1. The training provided me with sufficient knowledge before starting the treatments	9	3.56 ± 0.53	3, 4
2. The training provided me with sufficient skills before starting the treatments	9	3.67 ± 0.50	3, 4
3. I would have benefited from more training days	9	4.56 ± 0.73	3, 5
4. Fewer training days would have been enough	9	1.33 ± 0.71	1, 3
5. More practical training would have been necessary for me to feel adequately prepared	9	4.33 ± 0.87	3, 5
6. More preparatory assignments before training days would have been needed	9	2.89 ± 0.78	2, 4
7. I would have preferred more online training days	9	1.44 ± 0.73	1, 3
8: I would have preferred more in-person training days	9	4.89 ± 0.33	4, 5
9: I was given enough time to complete the training properly	9	3.56 ± 1.24	1, 5
10. I received the support and backing I needed from my workplace	9	4.33 ± 1.12	2, 5
11. I am satisfied with the quality of the training	9	3.89 ± 0.60	3, 5
12. I participated actively during the training	9	4.22 ± 0.67	3, 5
13. I feel adequately prepared for starting the treatments	9	3.11 ± 0.78	2, 4

*M* Mean, *SD* Standard Deviation

#### Need for practical training

When asked what they needed from the upcoming on-site training workshop, a majority of the facilitators expressed that they still felt they needed a better overview of the study and their tasks. They also expressed a need to practice the psychotherapeutic components of the treatment, regardless of how confident they felt about this part of the treatment: *“I don’t feel any concern about the psychotherapeutic part*,* but I hope to learn through experience-based learning - trying out being both patient and therapist.”* After finishing the on-site training workshop, most of the facilitators expressed that this part of the training had been particularly valuable, giving them a more coherent understanding and overview of the study and allowing them to develop their practical skills through the on-site exercises. One facilitator noted: *“Even though it may feel a bit unclear and uncertain before the start of the study*,* the workshop contributed significantly. It was extremely valuable to get to know - and practice with each other.”* The facilitators also indicated that they preferred the on-site training and found it more effective for learning compared to the online training. Still, they expressed a need for further practical training. As one facilitator noted: *“More practice rather than theory would probably have been helpful for the therapeutic parts.”*

#### Challenges during training

Some facilitators reported feeling overwhelmed by the training material and felt they needed more time to process it, while others indicated that additional training material would have been beneficial for them. When asked what was particularly difficult about the training, several facilitators expressed difficulties with gaining a sense of the ‘big picture’ and a need for more clarity on the structure of both the training and the overall study, particularly in the initial training phase. One facilitator also expressed difficulties with the role-play assessment, explaining that recording themselves had felt forced and unnatural.

#### Suggested improvement of training

Finally, one facilitator suggested having a nurse present to help educate in future training as a way to bridge the gap between fields. To improve the training, another facilitator suggested that the on-site training should be placed in the beginning rather than at the end of the training program: *“I wished that we had started with physical meetings at an earlier stage. I gained a completely different perspective that I maybe wished I had gotten earlier.”*

### The objective assessment of the facilitators’ verbal relational skills

After the correction for multiple comparisons, results of the Wilcoxon Signed Rank Test of the MITI-coded role-plays showed a significant increase in one of the two global scores (*Partnership*), but no significant change in any of the ten behavior counts pre- to post-training. The results also showed a large effect size for the global score *Empathy* and medium effect sizes for the global score *Partnership* and four of the behavior counts (*Affirm*, *Giving information*, and *Simple*- and *Complex reflection* (Table 3).

**Table 3:** Analyses of the CAPSI facilitators’ MITI scores pre- to post-training

	Pre-training			Post-training					
	*N*	*M*	*SD*	*N*	*M*	*SD*	*z*	*p*	*r*
Global Scores									
Partnership	9	2.11	0.33	9	2.56	0.53	-2.00	.046*	− .47
Empathy	9	1.22	0.44	9	1.89	0.60	-2.12	.068	− .50
Behavior Counts									
Affirm	9	0.00	0.00	9	0.33	0.50	-1.73	.830	− .40
Seeking collaboration	9	0.33	0.50	9	0.22	0.44	-1.00	.576	− .23
Emphasize autonomy	9	0.00	0.00	9	0.00	0.00	0.00		
Persuade	9	0.00	0.00	9	0.00	0.00	0.00		
Confront	9	0.00	0.00	9	0.00	0.00	0.00		
Giving information	9	21.44	3.09	9	19.44	3.25	-1.37	.573	− .32
Persuade with permission	9	0.00	0.00	9	0.11	0.33	-1.00	.576	− .23
Simple reflection	9	0.44	0.73	9	1.67	1.73	-1.56	.600	− .37
Complex reflection	9	0.22	0.44	9	0.67	0.71	-1.27	.515	− .30
Question	9	4.67	1.80	9	4.11	2.42	-0.43	.959	− .10

*M* = Mean; *SD* = Standard Deviation; *z* = the Wilcoxon Signed Rank Test statistic; *p* = probability value; *r* = Pearson’s Correlation Coefficient

*Significant at *p* < .05 after correction for multiple comparisons

## Discussion

This study described and evaluated the training of nurses as facilitators in the CAPSI trial. In the analysis of the subjective training evaluations, the facilitators’ answers to the free text questions largely reflected, but also somewhat complemented, the responses given in the online survey. The most prominent aspect of both evaluations was the facilitators’ preference for additional in-person, practical training. The assessment of their verbal relational skills, conducted with two standardized role-plays (pre- and post-training), showed a significant increase in one of the twelve MITI variables and medium to large effect sizes for six of the measures pre- to post-training (Table 3). Despite the medium to large effect sizes, most outcomes did not reach statistical significance. This is likely due to the small sample size and the use of the nonparametric Wilcoxon signed-rank test, both of which reduce statistical power, alongside the Benjamini–Hochberg procedure applied to correct for multiple comparisons.

### The subjective training evaluation

Most facilitators found the online training helpful in providing a general overview of the study, its treatment components, and their role as facilitators, including the required skills and competencies. They also felt well-supported by their workplace and reported participating actively in the training. Even so, many of the facilitators expressed a strong need for additional in-person and practical training to feel adequately prepared for the treatment.

In this study, a hybrid model combining both online and in-person training was employed to leverage the strengths of both formats. However, a large part of the training program consisted of webinars. While online training offers significant advantages such as cost-effectiveness, increased accessibility, and greater flexibility for trainees, it may also have certain limitations. Specifically, online training may not be optimal for training practical elements critical for the development of complex therapeutic skills, such as verbal relational skills [42]. This sentiment was also reflected in subjective evaluations of the training, where the facilitators specifically asked for further in-person training to develop their skills. The results of the subjective evaluations are consistent with previous findings, such as those of Tai et al. (2021). Here, trainees reportedly found the didactic and experiential interactive learning, as well as the clinical training and participant care under guidance of experienced therapists, to be the most valuable aspects of training. However, perceptions of training quality tend to favor in-person formats. A recent study found significantly higher ratings among trainees who receive in-person training compared to those who receive online training, despite no actual difference being observed in the overall effectiveness of the training [37]. Furthermore, a recent systematic review and metanalysis also found no significant difference when comparing the effects of training delivered online and training delivered in-person [31]. This suggests that while in-person training may improve confidence and perceived preparedness, the measured outcomes of training may not differ significantly based on the delivery method.

### The objective assessment of verbal relational skills

The significant increase in one of the twelve MITI variables and the medium to large effect sizes for six of the measures pre- to post-training (Table 3) indicates that the training was somewhat effective in strengthening the facilitators’ verbal relational skills when demonstrated in role-play. Although there were no significant changes in the behavior counts, most of these still tended in a positive direction. In addition, none of the facilitators engaged in either persuading or confronting during the role-plays, behaviors which can have a significant negative impact on the therapeutic relationship [43]. However, all MITI scores except for *Giving information* were low at baseline and remained low after the training (Table 3). While these scores may be due to factors such as the method and timing of assessment (see limitations below), several other factors may account for the results as well.

First, the low scores post-training may indicate that the training program was not effective enough. This could be due to a lack of both the intensity and duration of training. Previous research has found more intensive and extensive training models effective in facilitating therapists’ skill development [35, 36]. Still, the optimal balance of training components, content and duration in therapist training remains unclear. While the intensity of a training program seems important for the development of therapist skills, the time required to fully integrate skills may vary between trainees, depending on individual learning curves [44]. In this study, the training program spanned several months, included multiple components and totaled 37 h of training and learning content. While this suggests that the training was relatively intensive, the facilitators still expressed a need for additional training to feel fully prepared in the subjective evaluations. In addition to the modest increase in verbal relational skills, this may point to the need for a more comprehensive training model for facilitators in a trial like CAPSI.

Additionally, the training program might have benefited from more active training components. Extensive research has demonstrated the importance of active learning for effective skill development in therapist training [33–36]. In this training program, active learning was incorporated through components such as role-plays that modeled clinical scenarios. Several of the facilitators noted in their evaluation that the active training was more beneficial for their learning than the theoretical training. However, as described earlier, this sentiment may also be tied to the general preference for in-person training. While the topic of verbal relational skills was covered in the online training, the actual training of these skills was mostly done in-person through practical exercises at the on-site training workshop. As previously discussed, the lack of improvement in verbal relational skills may suggest that the balance between online and on-site components was not optimal to learn these skills. Since research has yet to show that in-person training is more effective than online training [31], this is merely speculation. It is more probable that the training program would have benefitted from additional active training components, such as role-play, in lieu of passive training components, such as lectures. And that these components should have been incorporated into the online training, not just the on-site training workshop.

Second, it is also worth considering whether the low scores in the facilitators’ verbal relational skills pre- and post-training are associated with their professional background. As described, all facilitators providing the psychotherapeutic support in the CAPSI trial are nurses. Nurses have played an important role in facilitating patient care in clinical trials since the beginning of modern psychedelic research [45] and have also been suggested as ideal psychedelic facilitators due to their professional sensibilities of caregiving [46]. However, given the nature of their work, nurses’ skills and competencies in caregiving may differ significantly from those of a psychotherapist. Nurses’ main task is to provide patient care by tending to the person’s whole being, both physically and psychologically. This requires them to have not only extensive medical knowledge and technical expertise, but also strong relational skills such as effective communication skills [47]. While communication is central to all forms of interpersonal care, it is used differently according to its aim and function within professions. Nurses are trained to convey critical health information, provide clear instructions, and offer emotional support to patients in a fast-paced work environment. Their communication tends to be concise, direct and practical, aimed at facilitating immediate care and decision-making [48]. In contrast, psychotherapists are trained to use communication as a tool to facilitate therapeutic processes, which demand a more dynamic and collaborative approach. A skilled psychotherapist will use questions and reflective listening to help patients explore their thoughts, feelings and circumstances, thereby fostering curiosity in the patient and allowing reflection and insight [49]. These differences in communication skills highlight a key distinction between the caregiving approaches of these two professions: While nurses are trained to address and resolve patients’ problems, psychotherapists are trained to facilitate collaborative therapeutic processes using deliberate techniques and interventions. The ability to explore, reflect and be present with a patient without attempting to “fix” the problem can be considered essential to the psychotherapeutic process [50], and differs substantially from the approach typical of nursing practice. Differences in caregiving competencies related to professional backgrounds were also recognized in the subjective evaluations of the training, where one facilitator suggested having a teacher with a background in nursing for future trainings to help educate and bridge the gap between professional fields. Additionally, challenges with the psychotherapeutic components of the treatment were expressed in the subjective evaluations: Prior to the on-site training workshop, several facilitators described feeling unsure of their own abilities to provide the treatment, and after completing the training, several also expressed a wish for further in-person training to gain more practical experience in this area.

### Limitations

This study has several limitations that may have influenced the results. First, as indicated by the behavior counts, the facilitators primarily provided information to the patient during the role-play (Table 3). While they may have been inclined to prioritize the delivery of information due to their professional background (as discussed above), it may also have been due to the method of assessment. Before each role-play, the facilitators were presented with written instructions for the role-play together with written information about the treatment. This may have led them to focus more on delivering accurate information to the patient, rather than relating to the patient in an empathic and collaborative manner. Furthermore, knowing they were being recorded and assessed as part of their training may have contributed to their low scores as well. While standardized role-play enables an equal and unbiased assessment of trainees [51], the method has also been critiqued for lack of ecological validity [52]. As one facilitator noted in the evaluation ‘being recorded felt unnatural and forced’. The facilitators possibly found it challenging to adequately demonstrate their verbal relational skills due to the awareness of simulation and the possible artificiality of the interaction during the assessment. Additionally, the role-play in this study involved two sets of instructions: before training, facilitators were asked to behave as they normally would, whereas after training, they were instructed to apply the verbal relational skills acquired.

Second, the low scores may have been due to the timing of the assessment. While the facilitators had completed their full training at the time of the second role-play, the supervision did not begin until after the entire training was completed, that is, after the second recording. Supervision plays a crucial role in developing therapeutic skills by providing structured feedback, opportunities for refinement of techniques, managing problem areas and fostering reflective practice [53]. Additionally, studies have shown that training and supervision is more effective compared to training alone when it comes to the improvement of therapist skills [30, 33, 54]. Thus, the facilitators’ verbal relational skills may have improved significantly since the post-training assessment as a result of the subsequent supervision. Future studies should consider incorporating assessments before and after both the training and the supervision to better capture the trajectory of skill development in training programs.

Third, the decision to only measure the facilitators’ verbal relational skills can be considered a substantial limitation in this study. The psilocybin treatment in the CAPSI trial comprises multiple elements, which the facilitators were trained in (e.g., GMVA). Including measurements of other areas of skill acquisition beyond the facilitators’ verbal relational skills may have given better insight into the effectiveness of the training.

Finally, the non-significant findings together with the medium to large effect sizes indicate that the small sample may have limited the chance to detect meaningful relationships in the collected data [55]. While this is a significant limitation, all the CAPSI facilitators were included as participants in this study. Studies with greater statistical power may be necessary to gain more knowledge in this research area. Relatedly, the broader applicability of this study is limited by its assessment of facilitators from a single professional background. Future studies should consider incorporating mixed cohorts to help clarify whether outcomes vary by professional background and assess the potential need for tailored training interventions.

## Conclusion

This exploratory study highlights both the promises and challenges of training facilitators for psilocybin treatments for depression. The findings indicate that the training model implemented in this study may have potential to improve facilitators’ verbal relational skills; however, changes were generally modest and only demonstrated in role-play. Additionally, although the facilitators reported that the training program supported their knowledge- and skill acquisition, they would have preferred additional practical training to feel adequately prepared. The precise requirements for the support surrounding the dosing in psilocybin treatments, including the specific skills and professional qualifications needed to provide it, remain unclear. Nonetheless, the results of this study suggest that different professionals may require distinct types of training to deliver these treatments effectively. As such, future clinical trials should design training programs based on the facilitators’ baseline skills by tailoring modules to address specific strengths and gaps in skills and competencies observed. Furthermore, to aid the development of facilitator training programs in clinical trials and potential future implementation in the healthcare system, future studies should provide clear descriptions and objective measures of the training interventions. Finally, future research is encouraged to replicate findings in controlled studies with larger and diverse samples, include longitudinal follow-up after supervision to capture the trajectory of skill development, and incorporate treatment adherence and competency assessments to ensure both fidelity and effectiveness of the treatment.

## Supplementary Information

Supplementary Material 1.

## Data Availability

The datasets used and analyzed in the current study are available from the corresponding author upon reasonable request.

## References

[1] Krebber AM, Buffart LM, Kleijn G, Riepma IC, de Bree R, Leemans CR, Becker A, Brug J, van Straten A, Cuijpers P, Verdonck-de Leeuw IM. Prevalence of depression in cancer patients: a meta-analysis of diagnostic interviews and self-report instruments. Psychooncology. 2014;23(2):121–30. 10.1002/pon.3409 PMC4282549 24105788

[2] Mejareh ZN, Abdollahi B, Hoseinipalangi Z, Jeze MS, Hosseinifard H, Rafiei S, et al. Global, regional, and national prevalence of depression among cancer patients: A systematic review and meta-analysis. Indian J Psychiatry. 2021;63(6):527–35. 10.4103/indianjpsychiatry.indianjpsychiatry_77_21 PMC8793718 35136248

[3] Brown KW, Levy AR, Rosberger Z, Edgar L. Psychological Distress and Cancer Survival: A Follow-Up 10 Years After Diagnosis. Psychosom Med. 2003;65(4):636–43. 10.1097/01.psy.0000077503.96903.a6 12883115

[4] Misono S, Weiss NS, Fann JR, Redman M, Yueh B. Incidence of Suicide in Persons With Cancer. J Clin Oncol. 2008;26(29):4731–8. 10.1200/JCO.2007.13.8941 PMC2653137 18695257

[5] Arrieta Ó, Angulo LP, Núñez-Valencia C, Dorantes-Gallareta Y, Macedo EO, Martínez-López D, et al. Association of Depression and Anxiety on Quality of Life, Treatment Adherence, and Prognosis in Patients with Advanced Non-small Cell Lung Cancer. Ann Surg Oncol. 2013;20(6):1941–8. 10.1245/s10434-012-2793-5 23263699

[6] Metaxa AM, Clarke M. Efficacy of psilocybin for treating symptoms of depression: systematic review and meta-analysis. BMJ. 2024;385:e078084. 10.1136/bmj-2023-078084 PMC11062320 38692686

[7] Grob CS, Danforth AL, Chopra GS, Hagerty M, McKay CR, Halberstadt AL, et al. Pilot Study of Psilocybin Treatment for Anxiety in Patients With Advanced-Stage Cancer. Arch Gen Psychiatry. 2011;68(1):71. 10.1001/archgenpsychiatry.2010.116 20819978

[8] Griffiths RR, Johnson MW, Carducci MA, Umbricht A, Richards WA, Richards BD, et al. Psilocybin produces substantial and sustained decreases in depression and anxiety in patients with life-threatening cancer: A randomized double-blind trial. J Psychopharmacol (Oxf). 2016;30(12):1181–97. 10.1177/0269881116675513 PMC5367557 27909165

[9] Ross S, Bossis A, Guss J, Agin-Liebes G, Malone T, Cohen B, et al. Rapid and sustained symptom reduction following psilocybin treatment for anxiety and depression in patients with life-threatening cancer: a randomized controlled trial. J Psychopharmacol (Oxf). 2016;30(12):1165–80. 10.1177/0269881116675512 PMC5367551 27909164

[10] Ross S, Agin-Liebes G, Lo S, Zeifman RJ, Ghazal L, Benville J, et al. Acute and Sustained Reductions in Loss of Meaning and Suicidal Ideation Following Psilocybin-Assisted Psychotherapy for Psychiatric and Existential Distress in Life-Threatening Cancer. ACS Pharmacol Transl Sci. 2021;4(2):553–62. 10.1021/acsptsci.1c00020 PMC8033770 33860185

[11] Studerus E, Kometer M, Hasler F, Vollenweider FX. Acute, subacute and long-term subjective effects of psilocybin in healthy humans: a pooled analysis of experimental studies. J Psychopharmacol (Oxf). 2011;25(11):1434–52. 10.1177/0269881110382466 20855349

[12] Carhart-Harris RL, Roseman L, Haijen E, Erritzoe D, Watts R, Branchi I, et al. Psychedelics and the essential importance of context. J Psychopharmacol (Oxf). 2018;32(7):725–31. 10.1177/0269881118754710 29446697

[13] Schlag AK, Aday J, Salam I, Neill JC, Nutt DJ. Adverse effects of psychedelics: From anecdotes and misinformation to systematic science. J Psychopharmacol (Oxf). 2022;36(3):258–72. 10.1177/02698811211069100 PMC8905125 35107059

[14] Horton DM, Morrison B, Schmidt J. Systematized Review of Psychotherapeutic Components of Psilocybin-Assisted Psychotherapy. Am J Psychother. 2021;74(4):140–9. 10.1176/appi.psychotherapy.20200055 34293927

[15] Beckman M, Poulsen S, Doss M, Stenbæk DS, Editorial. The psychotherapeutic framing of psychedelic drug administration. Front Psychol. 2023;14:1121234. 10.3389/fpsyg.2023.1121234 PMC9904325 36760432

[16] Goodwin GM, Malievskaia E, Fonzo GA, Nemeroff CB. Must Psilocybin Always Assist Psychotherapy? Am J Psychiatry. 2024;181(1):20–5. 10.1176/appi.ajp.20221043. Epub 2023 Jul 12. PMID: 37434509. 37434509

[17] Aday JS, Horton D, Fernandes-Osterhold G, O’Donovan A, Bradley ER, Rosen RC, et al. Psychedelic-assisted psychotherapy: where is the psychotherapy research? Psychopharmacology. 2024;241(8):1517–26. 10.1007/s00213-024-06620-x 38782821

[18] Chisamore N, Johnson D, Chen MJQ, Offman H, Chen-Li D, Kaczmarek ES, et al. Protocols and practices in psilocybin assisted psychotherapy for depression: A systematic review. J Psychiatr Res. 2024;176:77–84. 10.1016/j.jpsychires.2024.05.051 38850581

[19] Okiishi J, Lambert MJ, Nielsen SL, Ogles BM. Waiting for supershrink: an empirical analysis of therapist effects. Clin Psychol Psychother. 2003;10(6):361–73.

[20] Johns RG, Barkham M, Kellett S, Saxon D. A systematic review of therapist effects: A critical narrative update and refinement to review. Clin Psychol Rev. 2019;67:78–93. 10.1016/j.cpr.2018.08.004 30442478

[21] Heinonen E, Nissen-Lie HA. The professional and personal characteristics of effective psychotherapists: a systematic review. Psychother Res. 2020;30(4):417–32. 10.1080/10503307.2019.1620366 31122157

[22] Wampold BE, Owen J, et al. Therapist effects: history, methods, magnitude, and characteristics of effective therapists. In: Bergin and Garfield’s Handbook of Psychotherapy and Behavior Change, edited by Michael Barkham, John Wiley & Sons, Incorporated, 2021.

[23] Murphy R, Kettner H, Zeifman R, Giribaldi B, Kartner L, Martell J, et al. Therapeutic Alliance and Rapport Modulate Responses to Psilocybin Assisted Therapy for Depression. Front Pharmacol. 2022;12:788155. 10.3389/fphar.2021.788155 PMC9009076 35431912

[24] Moyers TB, Rowell LN, Manuel JK, Ernst D, Houck JM. The Motivational Interviewing Treatment Integrity Code (MITI 4): Rationale, Preliminary Reliability and Validity. J Subst Abuse Treat. 2016;65:36–42. 10.1016/j.jsat.2016.01.001 PMC5539964 26874558

[25] Rollnick S, Miller WR. What is Motivational Interviewing? Behav Cogn Psychother. 1995;23(4):325–34. 10.1017/S1352465809005128 19364414

[26] Phelps J. Developing Guidelines and Competencies for the Training of Psychedelic Therapists. J Humanist Psychol. 2017;57(5):450–87.

[27] Levin AW, Lancelotta R, Sepeda ND, Gukasyan N, Nayak S, Wagener TL, et al. The therapeutic alliance between study participants and intervention facilitators is associated with acute effects and clinical outcomes in a psilocybin-assisted therapy trial for major depressive disorder. Covington H, editor. PLOS ONE. 2024;19(3):e0300501. 10.1371/journal.pone.0300501 PMC10939230 38483940

[28] Roth AD, Pilling S, Turner J. Therapist Training and Supervision in Clinical Trials: Implications for Clinical Practice. Behav Cogn Psychother. 2010;38(3):291–302. 10.1017/S1352465810000068 20367895

[29] Tai SJ, Nielson EM, Lennard-Jones M, Johanna Ajantaival RL, Winzer R, Richards WA, et al. Development and Evaluation of a Therapist Training Program for Psilocybin Therapy for Treatment-Resistant Depression in Clinical Research. Front Psychiatry. 2021;12:586682. 10.3389/fpsyt.2021.586682 PMC7908919 33643087

[30] Henrich D, Glombiewski JA, Scholten S. Systematic review of training in cognitive-behavioral therapy: Summarizing effects, costs and techniques. Clin Psychol Rev. 2023;101:102266. 10.1016/j.cpr.2023.102266 36963208

[31] Ragnarsson EH, Reinebo G, Ingvarsson S, Lindgren A, Beckman M, Alfonsson S, et al. Effects of Training in Cognitive Behavioural Therapy and Motivational Interviewing on Mental Health Practitioner Behaviour: A Systematic Review and Meta-Analysis. Clin Psychol Psychother. 2024;31(3):e3003. 10.1002/cpp.3003 38855846

[32] Schwalbe CS, Oh HY, Zweben A. Sustaining motivational interviewing: a meta-analysis of training studies. Addiction. 2014;109(8):1287–94. 10.1111/add.12558 24661345

[33] Beidas RS, Kendall PC. Training therapists in evidence-based practice: A critical review of studies from a systems-contextual perspective. Clin Psychol Sci Pract. 2010;17(1):1–30. 10.1111/j.1468-2850.2009.01187.x PMC2945375 20877441

[34] Herschell AD, Kolko DJ, Baumann BL, Davis AC. The role of therapist training in the implementation of psychosocial treatments: A review and critique with recommendations. Clin Psychol Rev. 2010;30(4):448–66. 10.1016/j.cpr.2010.02.005 PMC2872187 20304542

[35] Rakovshik SG, McManus F. Establishing evidence-based training in cognitive behavioral therapy: A review of current empirical findings and theoretical guidance. Clin Psychol Rev. 2010;30(5):496–516. 10.1016/j.cpr.2010.03.004 20488599

[36] Frank HE, Becker-Haimes EM, Rifkin LS, Norris LA, Ollendick TH, Olino TM, et al. Training with tarantulas: A randomized feasibility and acceptability study using experiential learning to enhance exposure therapy training. J Anxiety Disord. 2020;76:102308. 10.1016/j.janxdis.2020.102308 PMC7680428 32992268

[37] Mallonee S, Phillips J, Holloway K, Riggs D. Training Providers in the Use of Evidence-Based Treatments: A Comparison of In-Person and Online Delivery Modes. Psychol Learn Teach. 2018;17(1):61–72.

[38] Messell C, Summer L, Bonde LO, Beck BD, Stenbæk DS. Music programming for psilocybin-assisted therapy: Guided Imagery and Music-informed perspectives. Front Psychol. 2022;13:873455. 10.3389/fpsyg.2022.873455 PMC9714440 36467212

[39] Moyers TB, Manuel JK, Ernst D. Motivational Interviewing Treatment Integrity Coding Manual 4.2. Unpublished Manual. 2014.

[40] Koo TK, Li MY. A Guideline of Selecting and Reporting Intraclass Correlation Coefficients for Reliability Research. J Chiropr Med. 2016;15(2):155–63. 10.1016/j.jcm.2016.02.012 PMC4913118 27330520

[41] Cohen J. Statistical power analysis for the behavioral sciences. 2nd ed. Hillsdale (NJ): Lawrence Erlbaum Associates; 1988.

[42] McMillen JC, Hawley KM, Proctor EK. Mental Health Clinicians’ Participation in Web-Based Training for an Evidence Supported Intervention: Signs of Encouragement and Trouble Ahead. Adm Policy Ment Health Ment Health Serv Res. 2016;43(4):592–603. 10.1007/s10488-015-0645-x 25822326

[43] Moeseneder L, Ribeiro E, Muran JC, Caspar F. Impact of confrontations by therapists on impairment and utilization of the therapeutic alliance. Psychother Res. 2019;29(3):293–305. 10.1080/10503307.2018.1502897 30047304

[44] Castonguay L, Boswell JF, Caspar F, Friedlander ML, Gómez B, Hayes AM, et al. What competencies should therapists acquire and how should they acquire them? In: Castonguay LG, Hill CE, editors. Becoming better psychotherapists: Advancing training and supervision. Washington: American Psychological Association; 2023 [cited 2024 Sep 30]. pp. 13–29. Available from: https://content.apa.org/books/17318-002.

[45] Denis-Lalonde D, Estefan A. Emerging Psychedelic-Assisted Therapies: Implications for Nursing Practice. J Ment Health Addict Nurs. 2020;4(1):e1–13.

[46] Penn AD, Phelps J, Rosa WE, Watson J. Psychedelic-Assisted Psychotherapy Practices and Human Caring Science: Toward a Care-Informed Model of Treatment. J Humanist Psychol. 2024;64(4):592–617.

[47] Peate I, Nair M, Wild K, Unit. The elements of care. Nursing practice. Volume 2. United Kingdom: John Wiley & Sons, Incorporated; 2014.

[48] Sibiya MN. Effective Communication in Nursing. In: Ulutasdemir N, editor. Nursing. InTech; 2018 [cited 2024 Oct 22]. Available from: http://www.intechopen.com/books/nursing/effective-communication-in-nursing.

[49] Hill CE, Norcross JC. Skills and Methods That Work in Psychotherapy: Research Results, Training Implications, Therapeutic Practices, and Task Force Conclusions. In: Hill CE, Norcross JC, editors. Psychotherapy Skills and Methods That Work. 1st ed. Oxford University PressNew York; 2023 [cited 2024 Sep 30]. pp. 676–702. Available from: https://academic.oup.com/book/46701/chapter/410467049. https://academic.oup.com/book/46701/chapter/410216131.

[50] Schneider K. Presence: The Core Contextual Factor of Effective Psychotherapy. Existential Anal. 2015;26(2):304–12.

[51] Marriott BR, Cho E, Tugendrajch SK, Kliethermes MD, McMillen JC, Proctor EK, et al. Role-Play Assessment of Therapist Adherence and Skill in Implementation of Trauma-Focused Cognitive-Behavioral Therapy. Adm Policy Ment Health Ment Health Serv Res. 2022;49(3):374–84. 10.1007/s10488-021-01169-9 34546482

[52] Liness S, Beale S, Lea S, Byrne S, Hirsch CR, Clark DM. Evaluating CBT Clinical Competence with Standardised Role Plays and Patient Therapy Sessions. Cogn Ther Res. 2019;43(6):959–70.

[53] Milne D. Evidence-Based Clinical Supervision: Principles and Practice. Wiley Blackwell; 2009.

[54] Rakovshik SG, McManus F, Vazquez-Montes M, Muse K, Ougrin D. Is supervision necessary? Examining the effects of internet-based CBT training with and without supervision. J Consult Clin Psychol. 2016;84(3):191–9. 10.1037/ccp0000079 26795937

[55] Fritz CO, Morris PE, Richler JJ. Effect size estimates: Current use, calculations, and interpretation. J Exp Psychol Gen. 2012;141(1):2–18. 10.1037/a0024338 21823805

