# Supplementary material for: Evaluation of a facilitator training program in a randomized controlled trial of psilocybin treatment for depression
Source: BMC Med Educ. 2026 Apr 9;26:635. doi: 10.1186/s12909-026-09124-8 (PMC13091270; doi:10.1186/s12909-026-09124-8)
Supplement: Supplementary file 1 — Supplementary Material 1. [file 12909_2026_9124_MOESM1_ESM.docx]

**Supplementary 1**

**Part one of the qualitative subjective training evaluation, conducted anonymously by the facilitators at the beginning of the on-site training.**

1. What do you take with you from the webinars?
2. What do you need from the workshop on-site?

**Part two of the qualitative subjective training evaluation, conducted anonymously by the facilitators at the end of the on-site training.**

1. How do you evaluate the on-site workshop and what do you take with you from this training?

**Part three of the qualitative subjective training evaluation, conducted online one week after the end of the full training period (i.e., five additional questions in free text format of an online survey evaluating the complete training program).**

Item 14: What was good about the training?

Item 15: What was less good about the training?

Item 16: Was there anything that was particularly difficult?

Item 17: Was there anything missing or that you would have liked more of?

Item 18: Is there anything else you would like to share about the training?
